# TALKING ABOUT LONELINESS

**DOI:** 10.1093/geroni/igz038.1368

**Published:** 2019-11-08

**Authors:** Michael Thomas

**Affiliations:** Brunel University London, Uxbridge, United Kingdom

## Abstract

his paper draws on insights from a mixed methods study on temporal variations in loneliness among older people (aged 50 years plus) in England. The paper highlights alternative strategies available to participants in research interviews, including engaging with personal experience of loneliness or avoidant tactics such as digression, projection onto others or claiming ignorance of the topic. The data point towards complicating factors including a reluctance to disclose loneliness as a socially undesirable phenomenon, as well as difficulties in articulating the complexities of this topic. These considerations remind researchers of the need for reflection on the use of qualitative interviews when investigating loneliness and other difficult aspects of personal experience.

